# Notum deacylates octanoylated ghrelin

**DOI:** 10.1016/j.molmet.2021.101201

**Published:** 2021-02-27

**Authors:** Yuguang Zhao, Laura-Nadine Schuhmacher, Morgan Roberts, Satoshi Kakugawa, Ganka Bineva-Todd, Steve Howell, Nicola O'Reilly, Christine Perret, Ambrosius P. Snijders, Jean-Paul Vincent, E. Yvonne Jones

**Affiliations:** 1 Division of Structural Biology, Wellcome Centre for Human Genetics, Oxford University, Oxford, OX3 7BN, UK; 2 Francis Crick Institute, London, NW1 1AT, UK; 3 Universite de Paris, Institut Cochin, INSERM, CNRS, F75014 Paris, France

**Keywords:** Notum, Ghrelin, Deacylation, Crystal structure, Metabolism

## Abstract

**Objectives:**

The only proteins known to be modified by O-linked lipidation are Wnts and ghrelin, and enzymatic removal of this post-translational modification inhibits ligand activity. Indeed, the Wnt-deacylase activity of Notum is the basis of its ability to act as a feedback inhibitor of Wnt signalling. Whether Notum also deacylates ghrelin has not been determined.

**Methods:**

We used mass spectrometry to assay ghrelin deacylation by Notum and co-crystallisation to reveal enzyme–substrate interactions at the atomic level. CRISPR/Cas technology was used to tag endogenous Notum and assess its localisation in mice while liver-specific Notum knock-out mice allowed us to investigate the physiological role of Notum in modulating the level of ghrelin deacylation.

**Results:**

Mass spectrometry detected the removal of octanoyl from ghrelin by purified active Notum but not by an inactive mutant. The 2.2 Å resolution crystal structure of the Notum-ghrelin complex showed that the octanoyl lipid was accommodated in the hydrophobic pocket of the Notum. The knock-in allele expressing HA-tagged Notum revealed that Notum was produced in the liver and present in the bloodstream, albeit at a low level. Liver-specific inactivation of Notum in animals fed a high-fat diet led to a small but significant increase in acylated ghrelin in the circulation, while no such increase was seen in wild-type animals on the same diet.

**Conclusions:**

Overall, our data demonstrate that Notum can act as a ghrelin deacylase, and that this may be physiologically relevant under high-fat diet conditions. Our study therefore adds Notum to the list of enzymes, including butyrylcholinesterase and other carboxylesterases, that modulate the acylation state of ghrelin. The contribution of multiple enzymes could help tune the activity of this important hormone to a wide range of physiological conditions.

## Highlights

• Mass spectrometry detects ghrelin deacylation using purified Notum.
• The crystal structure of the Notum-ghrelin complex is described.
• Knock-in mice show Notum in their liver and bloodstream.
• Liver-specific knock-out mice have increased acylated ghrelin in their circulation under a high-fat diet.

## 1 Introduction

Stomach-produced ghrelin is a 28 amino acid-secreted peptide with an eight carbon fatty acid covalently linked to the side chain oxygen atom of the third serine residue. Acylated ghrelin (AG) is a endogenous ligand for growth hormone (GH) secretagogue receptor-type 1a (GHS-1a) [1]. Activation of the receptor by acylated ghrelin (AG) potently stimulates GH secretion from the pituitary, increasing adiposity and reducing energy expenditure [2,3]. The octanoyl group is essential for this activity and, indeed, deacyl ghrelin (DAG) does not activate the GHS-R1a receptor [1]. Thus, to decipher the regulation of this metabolic pathway, it is essential to determine how ghrelin's lipidation state is regulated.

Ghrelin acylation is mediated by ghrelin O-acyltransferase (GOAT), a member of the membrane-bound O-acyl transferase (MBOAT) family [4,5]. While multiple potential enzymes that deacylate ghrelin have been identified, these are not fully defined [6]. Butyrylcholinesterase (BChE) [7], carboxylesterase [8], platelet-activating factor acetylhydrolase [9], and lysophospholipase 1 [10] have been proposed to act as ghrelin deacylases. Among these, BChE is one of the best characterised deacylases of choline and other small molecules and has also been shown to deacylate ghrelin. Purified BChE can remove octanoyl lipid from AG as demonstrated by mass spectrometry [11]. BChE knock-out mice exhibit slightly increased serum AG levels while re-introduction of BChE via viral infection can decrease the serum AG level [7]. BChE deficiency in mice promotes adipose tissue growth [12,13], and knock-out animals exhibit aggressive behaviour that may be linked to increased AG levels [14]. Although the BChE apo structure has been determined [15], structural information is not available for an enzyme-ghrelin or enzyme-octanoyl lipid complex. The BChE crystal structure shows that catalytic serine 198 is buried at the bottom of a narrow and deep pocket, which in its uncomplex state seems unlikely to be able to bind ghrelin lipids. However, molecular simulation modelling suggests that this pocket is sufficiently plastic to accommodate relatively bulky 8 carbon octanoyl lipids [14]. Thus, in combination, a number of studies have highlighted BChE as a deacylase that potentially contributes to ghrelin de-lipidation, although other enzymes are also likely to be involved.

We recently identified the Wnt antagonist Notum, a member of the α/β hydrolase superfamily, as a Wnt deacylase that can remove large (C16) palmitoleate lipids from Wnt ligands [16,17]. Importantly, Notum can also act on substrates bearing shorter lipids [16]. The structure of Notum in a complex with a 14-carbon myristoleic acid lipid has been reported, and Notum efficiently cleaves an 8-carbon-linked chromogenic p-nitrophenyl (pNP) ester substrate [16]. Indeed, OPTS (8-octanoyloxypyrene-1,3,6-trisulfonate) is a convenient fluorogenic substrate for *in vitro* Notum activity assays [[18], [19], [20], [21]]. These observations inspired us to investigate if Notum could deacylate octanoyl lipid-linked ghrelin, which is the only extracellular protein besides Wnts known to be modified by O-linked serine lipidation.

## 2 Materials and Methods

### 2.1 Synthesis of acylated ghrelin

The AG peptide tagged with biotin at the C-terminus was synthesised on an Activotec P11 Automated Peptide Synthesiser (Activotec, UK, Supplementary Information). The resulting peptide was comprised the following sequence: NH2-GS(S(octanoyl))FLSPEHQRVQQRKESKKPPAKLQPR-biotin. The calculated molecular weight (MW) was 3637.97.

### 2.2 Mass spectrometry

Synthetic biotinylated AG (1 μg in 4 μl) was mixed with 25 ng (in 1 μl) of Notum (Notum_core_, Ser 81-Thr 451, and Cys330Ser [16]) or an identical amount of an inactive mutant (S232A) protein in buffer containing 20 mM of ammonium bicarbonate. The mixture was incubated at 25 °C for 16 h and the reaction was quenched with 0.1% TFA. The samples were then desalted using a C18 Zip Tip. The samples were prepared in α-cyano-4-hydroxycinnamic acid in 50:50 water/acetonitrile with 0.1% TFA. MALDI-TOF spectra were acquired using an ABSCIEX 5800 TOF/TOF system and analysed using Data Explorer v4.11.

### 2.3 Notum and acyl-ghrelin co-crystallisation

Human AG (Sigma catalogue number G3902) was mixed in an equal molar ratio with purified human Notum_core_ (S232A) [16] at a concentration of 5 mg/ml. The crystals were grown using a ProPlex screen (Molecular Dimensions) D3 well (0.1 M of HEPES, pH 7, 0.1 M of KCl, and 15% PEG 5000 MME). The crystals were flash frozen by immersion in reservoir solution supplemented with 25% (v/v) glycerol followed by transfer to liquid nitrogen.

### 2.4 Data collection and structure determination

Datasets were collected using the Diamond Light Source (I04-1 beamline). Data images (exposure time 0.08 s) with a 0.1° rotation were recorded (total 180°) on a PILATUS 6M detector at a wavelength of 0.928 Å. The data were processed with Xia2 [22]. The structures were determined by molecular replacement (MOLREP [23]) with PDB 4UZ1 as a search model. The model was built with COOT [24] and refined with Phenix [25]. The data collection and refinement statistics are shown in Supplementary Table 1. The figures were prepared using PyMOL [26].

### 2.5 Animal studies

#### 2.5.1 Notum-HA knock-in mice

To generate a mouse strain expressing C-terminal HA-tagged Notum (Notum-HA) from the endogenous locus, we used a CAS9 guide gene-editing strategy (procedure performed by the genome modification platform at Crick Institute). Single-cell zygotes obtained from C57BL/6J (Charles River, Margate, UK) were micro-injected with a solution containing Cas9 (20 ng/μl; 0.06 μM), guide RNA targeting the genomic sequence 5′-TGCTGAGTAATGGGAACTAG-3′ (20n g/μl; 0.3 μM), and single-stranded repair DNA (20 ng/μl; 0.3 μM) in a buffer comprised of 5 mM of Tris-Cl and 0.1 mM of EDTA in ultrapure H_2_O with a pH of 7.4. The repair DNA sequence was: 5′-GCAGACGGTGGCTCAGCAGCAGGGGATGGAGCCCAGCAAGCTGCTAGGGATGCTGAGTAATGGGAACTACCCATACGACGTCCCTGACTATGCGGGGTATCCGTATGATGTGCCATACGCCTAGAGGGTGTGGTACTGAGGGGCTGGCCCACTCACTGCCCCAGGGTGACTCACCTCGACTTCTAGGTAC-3′. Cas9 and oligonucleotides were obtained from IDT (Leuven, Belgium). A total of 239 zygotes were transferred into recipient mice following injection, leading to the birth of 10 offspring with one MiSeq-confirmed hit, which was kept for further research.

#### 2.5.2 Liver-specific knock-out mice

Notum lox/lox mice on a C57BL6/J background [27] were crossed with B6.Cg-Speer6-ps1^Tg(Alb−Cre)21Mgn^/J (JAX stock number 003574, MGI: 2176228, referred to as Alb-Cre). The wild-type (WT) mice used in this study were mice lacking Alb-Cre obtained from the above cross. All of the procedures were carried out according to UK Home Office regulations at the Francis Crick Institute in London, UK. The animals were raised on a standard diet (SD) or high-fat diet (HFD) (Research Diet D12331, with 58 kcal% fat and sucrose) from weaning (approximately 20–21 days after birth). The mice were kept in individually ventilated cages on a 12-h/12-h dark/light cycle and had *ad libitum* access to food and water.

#### 2.5.3 Plasma sampling

Whole blood was obtained by cardiac punctures with a 25G needle under terminal anaesthesia. Whole blood was collected on ice in K3-EDTA (Sarstedt) microvettes with the addition of 10% HCL and 5 μM of methoxy arachidonyl fluorophosphonate (MAFP) to inhibit ghrelin deacylation during sample processing [28]. Plasma was separated from the cells by centrifugation at 2000× *g* for 15 min at 4 °C and stored at −80 °C until use.

### 2.6 Western blotting, immunoprecipitation, and immunofluorescence staining

Standard procedures were used for Western blotting detection of Notum-HA from mouse liver extract and plasma. HA-tagged mouse liver tissue sections were stained with anti-HA, anti-CD31 (Pecam-1), and anti-glutamine synthetase (GS), and fluorescent images were recorded with a Leica SP5 confocal microscope. The following antibodies were used: rabbit anti-HA (Cell Signalling catalogue number 3724S), mouse anti-glutamine synthetase (BD Biosciences catalogue number 610517), and rat anti-mouse CD31/Pecam-1 (BD Biosciences catalogue number 553370). Further experimental details are available in the Supplementary Methods.

### 2.7 Acyl ghrelin and deacyl ghrelin quantification

AG and DAG were quantified from plasma using a commercially available enzyme immunoassay kit according to the manufacturer's instructions (ghrelin mouse/rat kits, Bertin Pharma for acylated ghrelin #A05117.96 and deacylated ghrelin #A05118.96).

### 2.8 Data analysis and visualisation

All of the data were analysed using GraphPad Prism 8. Ghrelin enzyme-linked immunosorbent assay (ELISA) data were analysed using an ordinary two-way analysis of variance (ANOVA) with multiple comparisons and Tukey's correction to test the influence and interaction of genotype and diet.

## 3 Results

### 3.1 Mass spectrometry detection of ghrelin de-acylation by Notum

**Figure 1:**
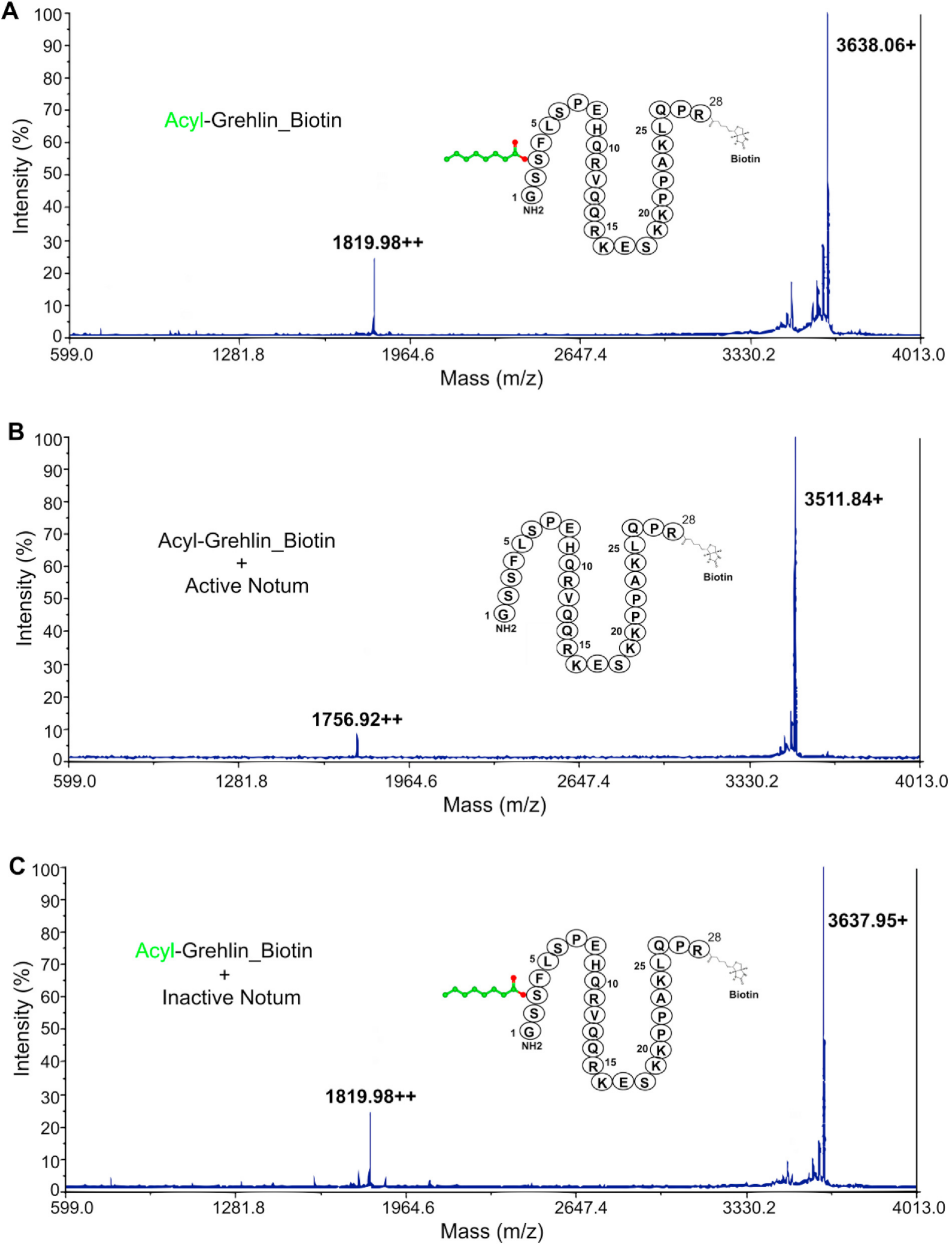
Mass spectra of AG treated with Notum. (A) The MALDI-TOF mass spectra of AG-biotin, with signal intensity (on the y axis) and *m*/*z* on the x axis. (B) Active Notum-treated AG-biotin. (C) Inactive Notum-treated AG-biotin.

To obtain direct evidence of ghrelin de-acylation by Notum, we used matrix-assisted laser desorption/ionisation time-of-flight (MALDI-TOF) mass spectrometry technology to detect mass changes after Notum treatment of synthetic biotinylated AG (AG-biotin; Methods). Active and inactive (S232A) Notum proteins were purified to homogeneity from HEK293T cells as previously described [16]. The AG-biotin had a mass-to-charge ratio (*m*/*z*) of 3638.06 with an additional double-charged peak of 1819.80 (Figure 1A), which agreed with the calculated mass of 3637.97 Da. When the peptide was treated with active Notum enzyme, the *m*/*z* shifted to 3511.84 (Figure 1B) with a double-charged peak of 1756.92. The loss of 126.13 Da matched well with an 8-carbon chain, indicating complete removal of the octanoyl lipids (Figure 1B). In contrast, when the inactive Notum mutant (S232A) enzyme was used, the mass of the untreated peptide was retained (Figure 1C). We therefore suggest that enzymatic activity is required for Notum to delipidate ghrelin.

### 3.2 Structure of acyl-ghrelin in complex with Notum

**Figure 2:**
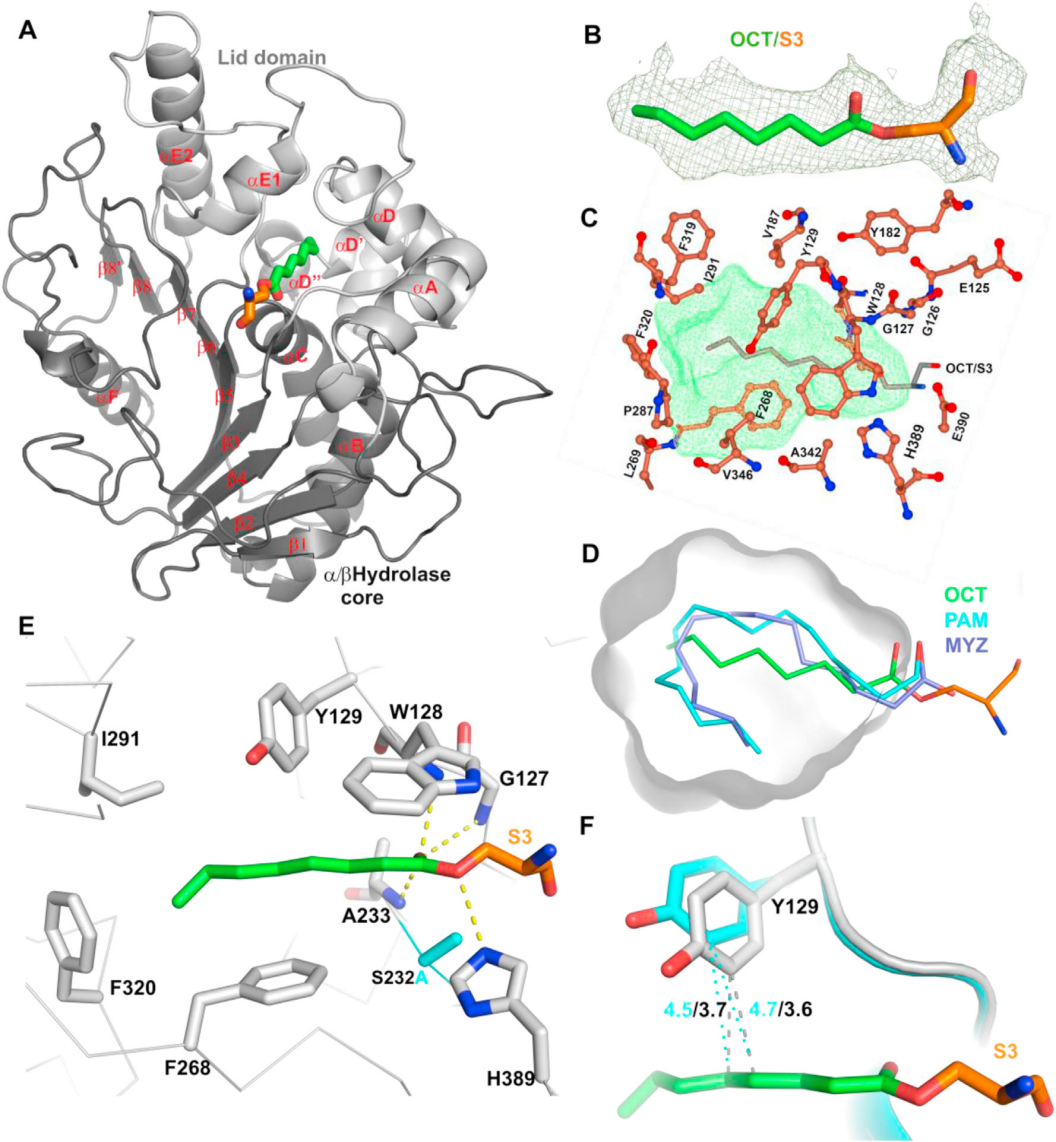
Structure of the Notum-ghrelin complex. (A) Cartoon presentation of the Notum-ghrelin complex structure. Notum is shown in cartoon (enzyme core in dark grey and lid domain in light grey); ghrelin serine-3 is shown as brown sticks with the octanoyl lipid coloured green. (B) |Fo–Fc| annealing omit electron density map contoured at 3 σ for the octanoyl lipid and ghrelin serine-3 (OCT/S3). (C) The Notum enzyme pocket is shown (green mesh) with surrounding residues denoted as brown sticks and balls. Octanoyl-linked ghrelin serine-3 is shown as grey sticks. (D) Comparison of the Notum enzyme pocket occupied with octanoyl (OCT, green), myristoleate (MYZ, light blue), and palmitoleic acid (PAM, cyan). The pocket is shown as a grey surface with 50% transparency. The cavity detection radius cut-off was set at 4 solvents. (E) Notum-ghrelin lipid interaction details. The Notum structure is shown as the Cα trace, the interaction residues are denoted as grey sticks (Ser 232 to Ala mutation in cyan), and the hydrogen bonds are represented by dashed yellow lines. (F) Comparison of the Notum residue Y129 side chain conformation in the Apo (cyan) and complex (grey) structures; hydrophobic interactions are shown as dashed lines.

To obtain the molecular details of the Notum-ghrelin interactions, we structurally analysed the co-crystals. As the active enzyme hydrolysed and released the octanoyl lipid from the ghrelin, we used the inactive mutant Notum_core_ (S232A) for co-crystallisation with AG. Crystals grew in a polyethylene glycol methyl ether (PEG MME) 5000 condition (Methods) and diffracted to a 2.2 Å resolution in space group P2_1_2_1_2 with two protomers in the crystallographic asymmetric unit (ASU). The structures of the two protomers were essentially identical to each other (rmsd 0.35 Å of all of the aligned 351 Cα). For simplicity, only one protomer complex is shown in Figure 2A. The overall Notum structure from the complex was similar to the reported apo structure [16] (PDB 4UZ1, rmsd 1 Å of aligned 345 Cα) and the complex with a lipidated Wnt7a peptide [16] (PDB 4UZQ, 0.65 Å of aligned 349 Cα). As a member of the α/β-hydrolase superfamily [29], the Notum's structure exhibited a typical core domain of β sheets wrapped by α helices (Figure 2A, dark grey) and a lid domain (Figure 2A, light grey). The lid domain adopted either an open or closed conformation [21]. The lid domain in the Notum-ghrelin complex had a closed conformation similar to the reported structure with the PDB code 4UYU [16]. Between the lid and core domains, extra electron density was observed, which was unambiguously modelled as octanoyl lipid linked to a serine residue via an ester bond (Figure 2B). The other residues from AG were disordered, as was the case for the Wnt7a peptide in our previous Notum complex structure, which only showed the palmitoleic acid lipid density [16]. The ghrelin octanoyl lipid sat centrally in the enzyme pocket. The Notum had a large hydrophobic enzyme pocket formed by residues including V187, E125 to Y129, Y182, L269, F268, P287, A290, I291, F319, F320, A342, and V346 as shown in Figure 2C. The pocket's volume was 379.5 Å³ as calculated by DoGSiteScorer [30]. Superimposition of the lipid from this complex with those from previously reported Notum complexes with 14 carbon myristoleic and 16 carbon palmitoleic acid lipids showed that the three lipids were well accommodated within the enzyme pocket (Figure 2D). The longer palmitoleic acid and myristoleic acid lipids were curved around the pocket, while the ghrelin octanoyl lipid adopted a linear conformation in the pocket's centre.

The interactions between the ghrelin octanoyl lipid and Notum were mainly hydrophobic. The lipid tail interacted with I291, F268, F320, and Y129, while the lipid head interacted with G127, W128, A233, and H389 (Figure 2E). Hydrogen bonds formed between the octanoyl acid and the backbones of G127, W128, and A233 and the side chain of H389 (Figure 2E). Compared with the apo Notum structure, all of the interacting residues maintained their side chain conformations, except Y129. The Y129 was repositioned to interact with the octanoyl lipid in the ghrelin complex (Figure 2F) with (the closest side chain carbon) distances of 3.6 and 3.7 Å to C4 and C5 of the lipid, respectively (for the superimposed apo structure, the equivalent distances were 4.7 and 4.5 Å, respectively, as shown in Figure 2F).

### 3.3 Notum liver expression and bloodstream secretion

**Figure 3:**
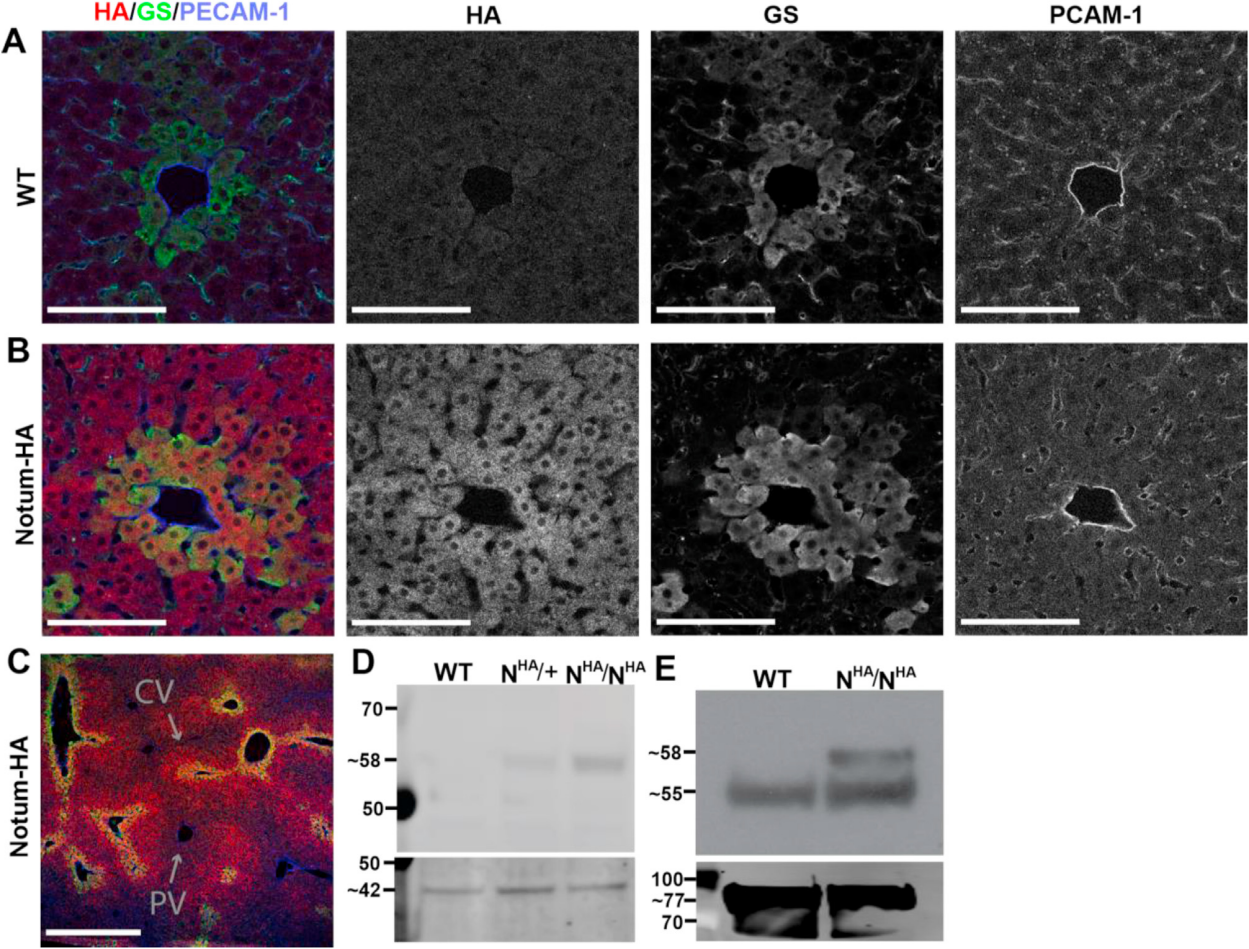
Immunofluorescence, Western blotting, and immunoprecipitation detection of Notum expression in the HA knock-in mice. (A) Liver section from a WT or (B) a homozygous Notum-HA mouse stained with anti-HA, anti-GS (terminal hepatic vein marker), and anti-PECAM-1 (endothelial cell marker). The scale bars represent 100 μm. (C) A low-magnification micrograph showing the central vein (CV) and portal vein (PV). The colour scheme is the same. The scale bar represents 500 μm. (D) Western blotting of mouse liver extracts stained with anti-HA. (E) Immunoprecipitation with HA antibody for mice plasma. WT, wild-type mice; N^HA^/+ and N^HA^/N^HA^ indicate heterozygous and homozygous Notum-HA knock-in mice, respectively. Molecular weight markers are shown in kDa. The bottom D and F panels show loading controls of β-actin and transferrin, respectively.

**Figure 4:**
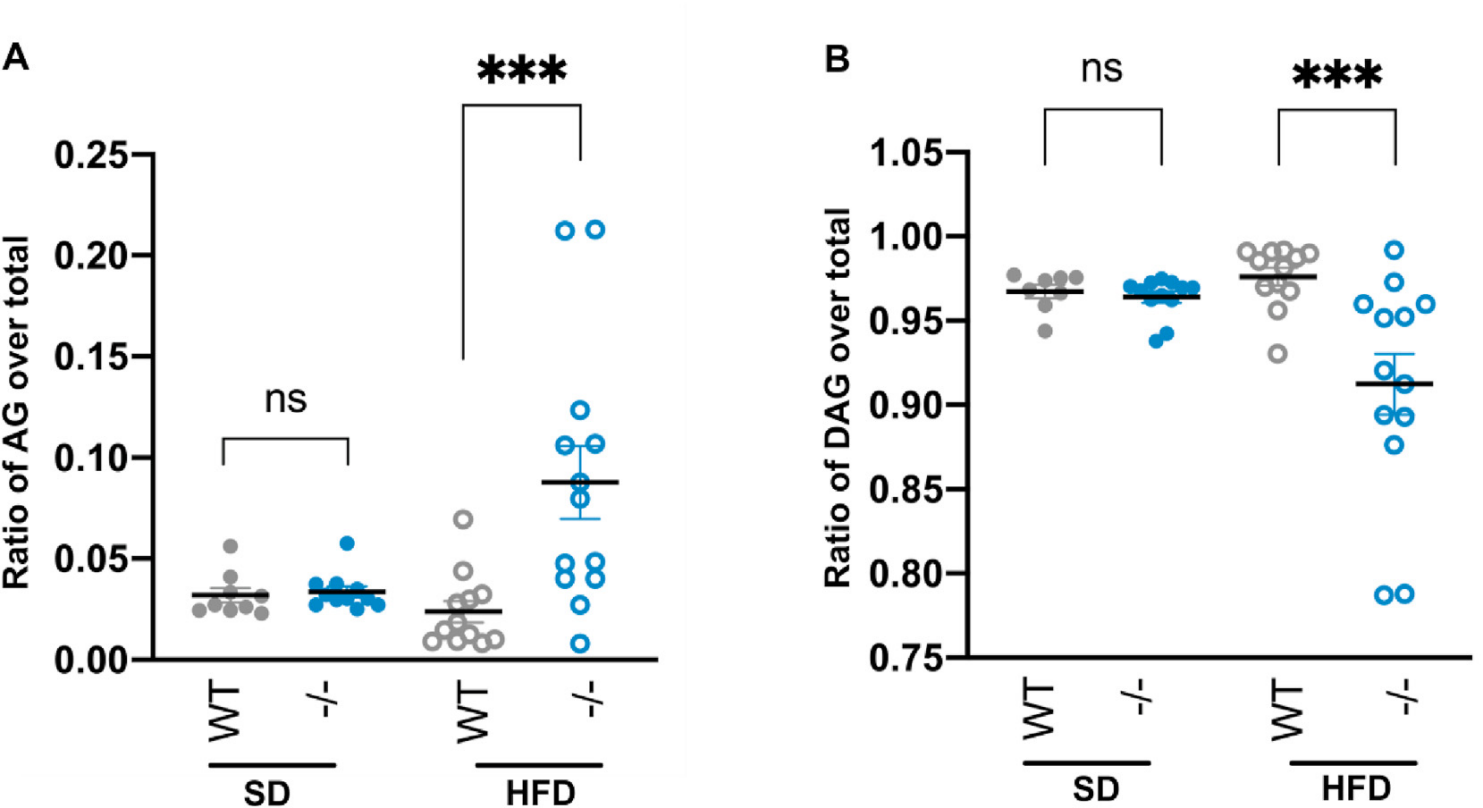
Comparison of mouse serum levels of ghrelin. (A) The ratio of AG over total ghrelin and (B) ratio of DAG over the total in the WT and liver-specific Notum knock-out mice (−/−) fed a SD or HFD. ∗∗∗ indicates statistical significance (P < 0.001) and ns indicates no statistically significant difference (P > 0.05).

Notum is highly expressed in the mouse liver as demonstrated by mRNA detection [27]. Detecting Notum protein was somewhat problematic because of the lack of fully validated antibodies. To detect endogenously expressed Notum protein, we used CRISPR/Cas technology [31] to knock DNA encoding an HA tag into the locus, thus creating a strain expressing C-terminally tagged Notum (Notum-HA, N^HA^). HA immunoreactivity was readily detected in liver sections of Notum-HA but not in the wild-type mice (Figure 3A,B). As expected from the localisation of mRNA, fluorescence was detected around the terminal hepatic vein (Figure 3C) marked by glutamine synthetase (GS) [32]. Hepatic vein localisation was further confirmed by staining with anti-PECAM-1 (platelet endothelial cell adhesion molecule-1, CD31), a specific endothelial cell marker (Figure 3A–C). We further performed Western blotting analysis, which revealed the presence of Notum in liver extracts from homozygous Notum-HA mice and to a lesser extent from heterozygotes, but not from unmodified mice (Figure 3D). To assess whether Notum was present in the blood, where ghrelin is normally found, we performed immunoprecipitation (IP) from serum with anti-HA. As shown in Figure 4E, a 55-kDa band, most likely corresponding to the heavy chain of the antibody used for immunoprecipitation, was detected in samples from both the WT and Notum-HA mice. A ∼58 KDa band, which matched the size of the Notum-HA, could be detected only in the samples from the Notum-HA mice (Figure 4E). These data showed that Notum protein is released into the bloodstream, likely from the liver and/or possibly from other organs.

### 3.4 Notum liver-specific knock-out mice increased blood acyl-ghrelin/total ratio on a high-fat diet

To determine whether Notum contributes to ghrelin deacylation *in vivo*, we assessed the proportion of AG and DAG in mice lacking specifically Notum in the liver (Notum^lox/lox^; Alb-Cre), which previously had been shown to display a mild metabolic phenotype [27]. In the first experiment, an ELISA assay was used to assess the level of AG and DAG in the mice fed a standard diet (SD). In wild-type animals, AG was a minor form (<5%, Figure 4) in agreement with earlier reports [33]. A similar figure was found in the Notum^lox/lox^; Alb-Cre mice (Figure 4), suggesting that under normal nutritional conditions, Notum did not impact ghrelin acylation. We then assayed the levels of AG and DAG in response to a high-fat diet (HFD), which was previously shown to affect ghrelin activity [[34], [35], [36]]. In this condition, the proportion of AG remained at a similarly low level in the WT controls. However, in the Notum^lox/lox^; Alb-Cre mice, this markedly increased and was accompanied by a corresponding decrease in the proportion of DAG. Therefore, Notum's deacylation activity might be particularly important for homeostasis of ghrelin acylation under metabolic stress caused by a HFD.

## 4 Discussion

Fatty acylation is an important post-translational modification of proteins, whereby fatty acids are covalently attached to proteins through amide or ester linkages to serine or thioester linkages to cysteine residues [6]. Although acylation usually targets cytoplasmic proteins, three classes of secreted proteins have been shown to undergo this modification: Hedgehogs, Wnts, and ghrelin [6]. Hedgehogs are acylated on an N-terminal cysteine, while Wnts and ghrelin are o-acylated on a conserved serine. Various enzymes, collectively known as the membrane-bound o-acyl transferase (MBOAT) family [37], are involved in the transfer of lipids onto these secreted proteins. Thus, Hhat transfers palmitate onto Hedgehogs, Porcupine (Porcn) appends a palmitoleate moiety in Wnt proteins, and another MBOAT (GOAT) adds a octanoyl group to ghrelin [4,5]. Importantly, Wnt acylation is reversible upon the activity of Notum, a secreted carboxylesterase that acts as a feedback inhibitor of Wnt signalling [16,17]. In this study, we asked whether Notum also deacylates ghrelin. Indeed, purified Notum reduced the mass of synthetic AG by 126 Da, consistent with the removal of an 8 carbon lipid. We determined the crystal structure, which showed at high resolution how this enzyme engages with the octanoyl lipid substrate. To the best of our knowledge, this is the first reported structure of ghrelin with a cognate deacylase.

Using a knock-in allele, we found that Notum is produced in the liver, which provides a route into the bloodstream, where it could control the acylation state of ghrelin. To test the physiological significance of this observation, we analysed the levels of AG and DAG in conditional mutant animals unable to produce Notum in the liver. Although no effect could be seen in normally fed animals, a significant increase in AG was detected under high-fat diet conditions, suggesting that Notum can modulate the acylation state of ghrelin. We note that, in knock-out mice lacking BChE, another ghrelin deacylase, similarly minor changes in AG and DAG levels were detected, along with an effect on food intake and glucose homeostasis [7]. Therefore, it appears that small changes in ghrelin's acylation state could be significant for the animals' physiology. A separate study showed that Notum^lox/lox^; Alb-Cre males are prone to accumulating pelvic fat from 12 months of age. This observation confirms the relevance of Notum in animal metabolism.

However, since Wnt signalling regulates fat [38] and glucose [39] metabolism, further research is needed to determine if Notum's metabolic effects are mediated primarily through ghrelin, Wnt, or both. To date, the general view has been that the link between Wnt signalling and metabolism is mediated by transcriptional targets of Wnt signalling, although other cross-talk mechanisms are possible (as reviewed by [40]). There is suggestive evidence that ghrelin could boost Wnt signalling [[41], [42], [43]], although the exact mechanisms have not been clearly defined. It is conceivable that in some circumstances, ghrelin could outcompete Wnt as a substrate for Notum, unleashing increased Wnt signalling. A competitive inhibition assay using the chromogenic pNP8 ester as a substrate and palmitoleic acid as an inhibitor suggested that the Wnt-associated C16 lipid could compete with the ghrelin-associated C8 lipid; however, there was no direct evidence for either being the strongly preferred Notum substrate [16]. Irrespective of substrate, the Notum is eminently druggable [18,21,[44], [45], [46]] and thus provides a potential route for pharmacological treatment of metabolic diseases.

## 5 Conclusions

We have provided biochemical and structural evidence showing that the Wnt-deacylating enzyme Notum can also deacylate ghrelin. The high–resolution complex structure revealed how ghrelin interacted with Notum. The knock-in HA-tagged Notum mice showed that Notum was expressed at the terminal hepatic vein and detectable in the blood. The liver-specific Notum knock-out mice had increased levels of AG. Taken together, our results suggest a new role for Notum in the regulation of ghrelin acylation.

## Data availablity

Data submitted to PDB with accession code 6ZYF.

## Funding

This study was funded by core funding of the 10.13039/100010438Francis Crick Institute (CRUK FC001204, MRC FC001204, and 10.13039/100010269Wellcome Trust FC001204), the 10.13039/501100000780European Union (ERC grants WNTEXPORT (294523) to JPV), 10.13039/501100000289Cancer Research UK (C375/A17721), the UK 10.13039/501100000265Medical Research Council (MR/M000141/1) to EYJ, and the 10.13039/100010269Wellcome Trust (203141/Z/16/Z) supporting the 10.13039/100004440Wellcome Centre for Human Genetics.

## Author contributions

YZ purified the Notum protein, performed the crystallisation, and determined the structure. L-NS conducted the mouse liver knock-out experiments and determined the ghrelin levels of the mice. MR analysed the N^HA^ mouse tissues and blood. NO'R and GB-T synthesised the acyl-ghrelin-biotin. SK initiated the study and performed the mass-spectrometry together with SH and APS. CP provided the Notum_loxP mice. YZ, JPV, and EYJ wrote the manuscript with contributions from L-NS and MR.
