# Supplementary material for: Notum deacylates octanoylated ghrelin
Source: Mol Metab. 2021 Feb 27;49:101201. doi: 10.1016/j.molmet.2021.101201 (PMC8010218; doi:10.1016/j.molmet.2021.101201)
Supplement: Multimedia component 1 [file mmc1.docx]

**Notum Deacylates Octanoylated Ghrelin**

**Supplementary methods**

**Synthesis of acylated ghrelin-biotin**

AG-biotin peptide was synthesised on an Activotec P11 Automated Peptide Synthesiser (Activotec, UK) on Universal NovaTag Resin (N-Fmoc-N'-Mmt-ethylenediamine-MPB-AM resin) using N(a)-Fmoc amino acids and HCTU as the coupling reagent. Other amino acids used were N(a)-Fmoc-Glu(tBu)-Ser(ψMe,Mepro)-OH, N(a)-Fmoc-Leu-Ser(ψMe,Mepro)-OH, N(a)-Fmoc-Ser-OH (at the site of octanoyl modification), and Boc-Gly-OH. The synthesis was performed at 60°C, except for coupling of His(Trt) and the final three amino acids. All the amino acids were double coupled, except for Fmoc-Ser-OH and Boc-Gly-OH. Following the amino acid chain assembly, octanoylation of the peptidyl resin was achieved as follows: octanoyl chloride (20 eq and 0.6 mM) was dissolved in 4 ml dimethylformamide (DMF), and the solution was added to the peptidyl resin (0.03 mM). Then, 4-dimethylaminopyridine (DMAP, 0.2 eq, and 6 µM) was added to 1.5 ml of pyridine, and this solution was also added to the peptidyl resin. The peptidyl resin was gently agitated for 2 h at room temperature (RT). The resin was washed with 3 x DMF and then with 2 x dichloromethane (DCM). The Mmt group was removed by treating the DCM swollen resin with 0.6 M of HOBt in DCM/TFE (1:1) for 3 h during which the solution became orange. The solution was removed by filtration, and the resin was washed with DCM. The treatment was repeated. Biotin was added to the newly exposed amino group at the C terminal as follows: biotin (98 mg) was dissolved in 1 mL of DMSO:NMP (1:1) activated with 400 µl of 1 M of HOBt in NMP for 3 min, and then 100 µL of DIC was added. After 30 min, this solution was added to the peptidyl resin (preswollen with DMF) and reacted for 16 h with gentle agitation. The resin was washed with 3 x DMF and then with 2 x DCM.

The peptide was cleaved from the resin by adding a cleavage cocktail (10 mL, 95% trifluoroacetic acid (TFA), 2.5% H_2_O, 2.5% triisopropylsilane (TIS) for 4 h. Following resin removal, peptide precipitation, and washing with ether, the peptide was dissolved in water solution and lyophilised overnight. A portion of the peptide was purified on a C8 reverse phase HPLC column (Agilent PrepHT Zorbax 300SB-C8, 21.2 x 250 mm, and 7 min) using a linear solvent gradient of 10-55% MeCN (0.08% TFA) in H_2_O (0.08% TFA) over 40 min at a flow rate of 8 mL/min. The resulting peptide had the following sequence: NH2-GS(S(octanoyl))FLSPEHQRVQQRKESKKPPAKLQPR-bio, with a calculated MW of 3637.97.

**Antibodies**

**Primary antibodies**

Anti-HA and anti-β-actin antibodies were purchased from Cell Signalling Technology (London, UK). Anti-CD31(Pecam-1) and anti-glutamine synthetase were obtained from BD Transduction Laboratories (Oxford, UK). Anti-transferrin antibody was acquired from Abcam (Cambridge, UK).

| Application | Antibodies | Dilution (in PBST) | Species | Clone | Company |
| --- | --- | --- | --- | --- | --- |
| WB | Anti-transferrin | 1:10000 | Rabbit | ab82411 | Abcam |
| WB and IF | Anti-HA | 1:1000 (WB and IF) | Rabbit | C29F4 | Cell Signalling Technology |
| WB | Anti-β-actin | 1:1000 | Rabbit | 4967L | Cell Signalling Technology |
| IF | CD31 (Pecam-1) | 1:200 | Rat | MEC 13.3 | BD Biosciences |
| IF | Anti-glutamine synthetase | 1:1000 | Mouse | 6 | BD Biosciences |

**Secondary antibodies**

Alexa Fluor Plus 488, Alexa Fluor 555, and Alexa Fluor Plus 647 were purchased from Thermo Fisher Scientific (London, UK). IRDye 800CW donkey anti-rabbit IgG was purchased from LI-COR Biosciences UK (Cambridge, UK). Alexa Fluor 680 IgG fraction monoclonal mouse anti-rabbit IgG light chain specific was purchased from Jackson ImmunoResearch Europe (Cambridge, UK).

| Application | Antibodies | Host | Target Species | Dilution in PBST | Clone | Company |
| --- | --- | --- | --- | --- | --- | --- |
| IF | Alexa Fluor Plus 488 | Goat | Mouse | 1:600 | A32723 | Thermo Fisher Scientific |
| IF | Alexa Fluor 555 | Goat | Rat | 1:600 | A-21434 | Thermo Fisher Scientific |
| IF | Alexa Fluor Plus 647 | Goat | Rabbit | 1:600 | A32733 | Thermo Fisher Scientific |
| WB | IRDye 800 CW | Donkey | Rabbit | 1:10,000 | 926-32213 | LI-COR Biosciences |
| WB | Alexa Fluor 680 IgG, light chain specific | Mouse | Rabbit | 1:400 | 211-622-171 | Jackson ImmunoResearch |

**Western blotting and immunoprecipitation**

All the samples were collected between 12:30 and 14:30. Mice (8-10 weeks old) were terminally anaesthetised using a pentobarbital intra-epicaine cocktail injected intra-peritoneally (IP)*.* After establishing a loss of the pedal-toe reflex by performing a pedal-toe pinch, the mice were crucified on a polystyrene culling board. The heart’s location was indicated by a light touch using a forefinger on the mouse’s left thoracic cavity. Blood was extracted directly from the heart using a 1 mL syringe after syringe penetration under the rib cage. Blood was collected in an EDTA/KE 1.3 tube (Sarstedt, Leicester, UK) to prevent coagulation and stored on ice. Following blood extraction, the thoracic cavity was opened, and the liver was extracted. The left lobule was dissected and washed in ice-cold PBS before being snap frozen in liquid nitrogen. Plasma was separated from serum using a microcentrifuge spun at 2,000 g rpm at 4°C for 15 min. Plasma and liver tissue were stored at -80°C until further use.

To detect Notum-HA in the plasma and liver extracts, an immunoprecipitation assay was performed using anti-HA magnetic beads (Thermo Fisher Scientific, Waltham, MA, USA). Before immunoprecipitation, the liver tissue was lysed in 500 μL Pierce IP lysis buffer (Thermo Fisher Scientific, Waltham, MA,USA). The protein concentration of the plasma and liver samples was measured using a Pierce BCA protein assay kit (Thermo Fisher Scientific, Waltham, MA, USA). Once the protein concentration was established, the liver and plasma samples were diluted in IP lysis buffer to 5 mg/mL. Then 40 μL of this dilution was added to 25 μL of 4 x BOLT LDS sample buffer (Thermo Fisher Scientific, Waltham, MA, USA), 10 μL of 10 x BOLT sample-reducing agent (Thermo Fisher Scientific, Waltham, MA, USA), and 25 μL of IP lysis buffer, leaving a 2 mg/mL sample for use as the input in Western blotting assay. This was heated to 85°C for 10 min in an incubator and stored at -20°C until further use. The remaining 5 mg/mL sample was used for the immunoprecipitation assay. Then, 40 μL of Pierce anti-HA magnetic beads was washed in 400 μL of IP lysis buffer three times. Next, 800 μL of the sample was added to the washed beads and left to incubate at 4°C on a rocker for 12 h. Then, 40 μL of the supernatant underwent the same protocol as described for the input. The remaining magnetic beads were washed three times in TBS-Tween 0.05%. The following was then added to the washed beads: 12.5 μL of 4 x BOLT LDS sample buffer (Thermo Fisher Scientific, Waltham, MA, USA), 5 μL of 10 x BOLT sample-reducing agent (Thermo Fisher Scientific, Waltham, MA, USA), and 32.5 μL of IP lysis buffer. This was heated to 85°C for 10 min in an incubator and either used directly or stored at -20°C until further use.

Following immunoprecipitation, 40 μL of input, supernatant, and IP product was fractioned by BOLT Bis-Tris Plus gel (Thermo Fisher Scientific, Waltham, MA, USA) and transferred to polyvinylidene difluoride (PVDF) membranes using a Mini Blot Module (Thermo Fisher Scientific, Waltham, MA, USA). Following transfer, the membranes were blocked with 5% non-fat milk in TBS-T for 60 min at RTP on a rocker, and then incubated with primary antibodies against anti-HA, anti-transferrin (for plasma samples), and anti-β-actin (for the liver samples) at 4°C for 12 h on a rocker. The membranes were washed three times for 10 min and incubated with a 1:600 dilution of secondary antibodies for 2 h. Blots were washed with TBS-T three times for 10 min and developed using an Odyssey CLx (LI-COR Biosciences UK, Cambridge, UK).

**Immunofluorescence**

The mice were anaesthetised as previously described and then sprayed with 70% ethanol before opening the thoracic cavity. The diaphragm was cut loose, and the rib cage opened and pinned away from the heart and lungs, exposing the heart. The right atrium was snipped using micro-scissors, and the left ventricle was punctured with a 23G needle (attached to tubing connected to a MINIPULS peristaltic pump (Gilson Scientific, Middleton, WI, USA). The mice were perfused with chilled phosphate-buffered saline (PBS) on ice at a constant rate of 4 mL/min for 8 min until all the plasma was cleared (no visible plasma leaving the heart). Following perfusion, the liver’s left lobe was extracted and dissected into 10 mg strips. The tissue strips were then washed in ice-cold PBS and placed in 4% paraformaldehyde (PFA) in PBS at 4°C for 2 h before immersion in 30% sucrose at 4°C for 12 h. The tissues were then embedded in optimal cutting temperature (OCT) and snap frozen in isopentane on dry ice. The OCT embedded block was stored at -80°C until further use. Before sectioning, the OCT-embedded tissues were allowed to equilibrate to the cryostat temperature for 1 h. Then, 12 μm liver sections were cut and fixed to slides. The slides were subsequently placed on ice and stored at -80°C until further use.

Before staining, the slides were allowed to thaw at room temperature for 30 min and then placed in chilled PBS for 30 min to permeabilise the tissue. All the subsequent steps were at room temperature. The tissue was blocked in 5% goat serum in 0.3 M of glycine PBS-Tween 0.1% and 0.05 M of calcium (PBS-TC) for 1 h. This was followed by incubation in the primary antibody diluted in PBS-TC at 4°C for 12 h. The primary antibody was washed 2 times for 5 min in high-salt PBS and then 10 min in PBS-TC. The tissue was then incubated in the secondary antibody solution (1:400 in PBS-TC) for 2 h and then washed as previously described. All of the samples were stained with Hoechst before mounting. All the immunofluorescence images were acquired with a 10 x (dry) or 63 x (oil immersion) objective on a Leica SP5 confocal microscope. Confocal images were processed with Volocity (PerkinElmer), ImageJ (NIH), and Illustrator (Adobe).

Scan settings

| **Objective** | **Scan speed (Hz)** | **Pinhole [m] (µm)/[airy] (µm)** | **Refraction index** | **Laser** | **PMT** | **Emission (nm)** | **Colour** | **Primary fluorophore** |
| --- | --- | --- | --- | --- | --- | --- | --- | --- |
| PL FLUOTAR 10.0 x 0.30 dry | 400 | 70.7/1.0 | 1.0 | Argon/20% (488 nm) | 1 | 518-585 | Green | Alexa Fluor Plus 488 |
|  |  |  |  | DPSS (561 nm) | 2 | 580-642 | Blue | Alexa Fluor 555 |
|  |  |  |  | HeNe (633 nm) | 3 | 654-737 | Red | Alexa Fluor Plus 647 |
| HCX PL APO Lambda Blue 63.0 x 1.40 oil UV | 400 | 95.5/1.0 | 1.52 | Argon/20% (488 nm) | 1 | 518-585 | Green | Alexa Fluor Plus 488 |
|  |  |  |  | DPSS (561 nm) | 2 | 580-642 | Blue | Alexa Fluor 555 |
|  |  |  |  | HeNe (633 nm) | 3 | 654-737 | Red | Alexa Fluor Plus 647 |

Laser modulation by acousto-optical tuneable filters (AOTF) and acousto-optical beam splitters (AOBS).

**Supplementary Table 1**: **Data collection and refinement statistics**

| **PDB ID** | 6ZYF |
| --- | --- |
| **Data collection** |  |
| X-ray source (Diamond) | I04-1 |
| Wavelength (Å) | 0.92819 |
| Space group | P2_1_2_1_2 |
| Cell dimensions |  |
| a, b, and c (Å) | 75.4, 184.1, and 61.8 |
| α, β, and γ (°) | 90, 90, and 90 |
| Resolution (Å) | 2.19 (2.23-2.19) * |
| *R*_merge_ | 0.086 (---) |
| *I* / σ*I* | 10.3 (1.2) |
| *CC 1/2* | 0.999 (0.527) |
| Completeness (%) | 100 (98.7) |
| Redundancy | 6.1 (5.0) |
| **Refinement** |  |
| Resolution (Å) | 47.81-2.19 |
| No. unique reflections | 45353 (2207) |
| *R*_work_/*R*_free_ | 0.220/0.259 |
| No. atoms |  |
| Protein (chain A/B) | 2811/2821 |
| Ligand/other | 25/36 |
| Water | 61 |
| *B* factors (Å^2^) |  |
| All atoms | 56.7 |
| Ligand | 60.8 |
| Rms deviations |  |
| Bond lengths (Å) | 0.007 |
| Bond angles (°) | 1.027 |

*Values in parentheses are for the highest resolution shell.
